# Clinical validation of an artificial intelligence model for thyroid fine-needle aspiration biopsy indication: comparison with TI-RADS systems and human specialists in a Chilean public hospital

**DOI:** 10.3389/fendo.2026.1809473

**Published:** 2026-05-11

**Authors:** Cristina Goens, Camilo Apey, Tomás San Martín, Nicole Bustíos, María Jesús Lira, Nikita Pozdeyev

**Affiliations:** 1 Endocrinology Department, Hospital Dra. Eloísa Díaz, Santiago, Chile; 2 Department of Internal Medicine, Faculty of Medicine, Universidad Finis Terrae, Santiago, Chile; 3 Radiology Department, Hospital Dra. Eloísa Díaz, Santiago, Chile; 4 Department of Radiology, Clínica Alemana de Santiago, Santiago, Chile; 5 Department of Medical Oncology, Clínica Santa María, Santiago, Chile; 6 Department of Orthopedics and Traumatology, Pontificia Universidad Católica de Chile, Santiago, Chile; 7 Department of Biomedical Informatics, Division of Endocrinology, Diabetes and Metabolism, University of Colorado School of Medicine, Aurora, CO, United States

**Keywords:** Artificial intelligence, biopsy, fine-needle, diagnostic imaging, risk assessment, thyroid nodule

## Abstract

**Introduction:**

Ultrasound evaluation of thyroid nodules shows significant interobserver variability, contributing to the overutilization of fine-needle aspiration biopsy (FNA). Standardized systems such as TI-RADS aim to reduce this variability; however, their performance in real-world clinical practice remains heterogeneous. Artificial intelligence (AI) has emerged as a potential tool to support clinical decision-making in this setting.

**Objective:**

To evaluate the clinical performance of an artificial intelligence model for recommending FNA in thyroid nodules and to compare it with ACR TI-RADS and Horvath TI-RADS classifications applied by specialists.

**Materials and methods:**

A retrospective cross-sectional study evaluating the clinical performance of an artificial intelligence model for FNA recommendation was conducted in adult patients who underwent thyroid ultrasound and FNA between January and December 2021. FNA recommendations generated by an AI model were compared with those issued by an expert radiologist and an expert endocrinologist using ACR TI-RADS and Horvath TI-RADS systems. The reference standard was cytology according to the Bethesda System, considering Bethesda ≥ III as a positive result, as an operational definition aligned with clinical decision-making for further diagnostic evaluation. Diagnostic performance metrics and concordance were calculated.

**Results:**

A total of 101 patients were included (89.1% women), with a median age of 61 years and a median nodule size of 2.3 cm (IQR: 1.6–3.3). The AI model showed a sensitivity of 0.88 and a specificity of 0.41. Horvath TI-RADS, applied by an expert radiologist, demonstrated a sensitivity of 0.82 and a specificity of 0.69. Concordance between AI and TI-RADS–based methods were moderate.

**Conclusion:**

In a real-world clinical setting, the AI model demonstrated performance comparable to that of human specialists for recommending FNA in thyroid nodules. These findings support its potential role as a complementary decision-support tool, particularly in settings with variability in ultrasound interpretation.

## Introduction

Thyroid nodules are a common finding in clinical practice, with a reported prevalence of up to 67% in the adult population, particularly among women and older individuals (1). Although approximately 95% of these nodules are benign, the widespread use of imaging studies—especially ultrasound—has significantly increased their incidental detection (2, 3). This phenomenon has been associated with a sustained increase in fine-needle aspiration biopsy (FNA), with relevant clinical, psychological, and economic implications for patients and healthcare systems, without a proportional reduction in thyroid cancer mortality (4).

Even though systematic ultrasound screening of the thyroid gland is not recommended by international or national clinical guidelines, thyroid ultrasound is widely used in routine clinical practice (5–8). Its interpretation is highly operator-dependent and shows significant interobserver variability, even among trained specialists, particularly in the assessment of key ultrasound features such as margins, echogenicity, and the presence of microcalcifications (9). The accurate assessment of these key features allows for cancer risk estimation of thyroid nodules and strongly impacts management (10). This interpretive heterogeneity results in substantial differences in FNA indication for similar ultrasound findings, contributing to unnecessary procedures and increased use of healthcare resources (11, 12).

Standardized ultrasound classification systems, such as TI-RADS, have been developed to reduce this variability and support biopsy indication through structured criteria. However, even with the application of these systems, relevant interobserver variability persists, especially in real-world clinical practice. In this context, AI systems are increasingly being assessed in thyroidology for their ability to support information interpretation and clinical communication (13), and particularly for thyroid nodules AI can be used to standardize ultrasound interpretation and FNA indication, without constituting a definitive diagnostic method for thyroid malignancy (14, 15). Nevertheless, evidence regarding its clinical performance in real-world healthcare settings remains limited (16, 17). Therefore, the aim of this study was to evaluate the clinical performance of an artificial intelligence model for fine-needle aspiration biopsy (FNA) recommendation and to compare it with ACR TI-RADS and Horvath TI-RADS systems applied by human specialists in a public hospital within the Chilean healthcare system. This study focuses on the evaluation of these tools in the context of clinical decision-making for FNA indication, rather than on the definitive diagnosis of thyroid malignancy, using Bethesda cytology (Bethesda ≥ III) as an operational reference standard.

## Materials and methods

### Study design and population

A retrospective cross-sectional diagnostic accuracy study was conducted in a tertiary public hospital within the Chilean healthcare system.

The study was approved by the Institutional Scientific Ethics Committee (Servicio de Salud Metropolitano Sur Oriente Comité Ético-Científico), with waiver of informed consent due to its retrospective nature and use of coded data.

The sample was selected by convenience, consistent with the retrospective nature of the study and the availability of complete ultrasound records and corresponding cytological results.

Adult patients (≥18 years) with a diagnosis of thyroid nodule evaluated by cervical ultrasound and who underwent fine-needle aspiration biopsy (FNA) between January and December 2021 were included. Patients with incomplete or poor-quality ultrasound images, images that could not be processed by the artificial intelligence model, duplicate records, and those with persistent Bethesda I cytology after repeated evaluation were excluded. All examinations were performed following standardized international acquisition protocols, (longitudinal and transverse planes, as well as color Doppler imaging). These studies were acquired using high-quality ultrasound systems (Philips iU22) and all images were stored and analyzed in high-quality DICOM format, preserving full image resolution.

**Figure 1:**
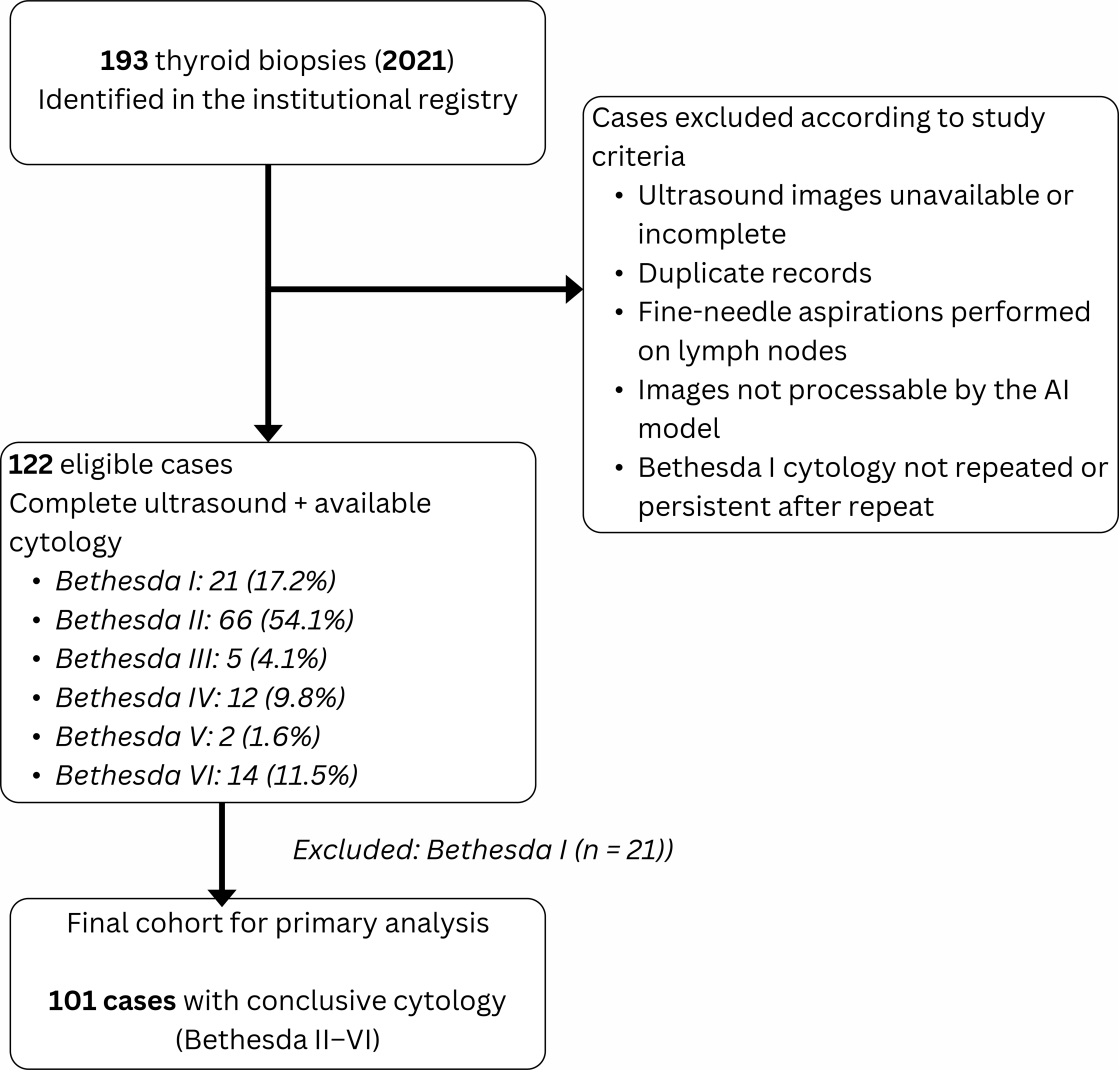
STROBE diagram. The study cohort was derived from 193 thyroid biopsies performed in 2021. A total of 122 cases met eligibility criteria (complete ultrasound and cytology). Cases with Bethesda I cytology were excluded, resulting in a final cohort of 101 nodules with conclusive cytology (Bethesda II–VI) used for the primary analysis.

The patient inclusion and exclusion flow are presented in a STROBE diagram (**Figure 1**). This study is reported in accordance with the STROBE guidelines for observational studies.

### Ultrasound procedure and FNA

Thyroid ultrasounds were performed as part of routine clinical practice using high-resolution equipment and high-frequency linear transducers. Fine-needle aspiration biopsies (FNA) were performed using an aspiration technique with 21G needles, following current institutional protocols. Given the clinical nature of the study, procedures were performed by different operators with variable levels of experience, reflecting a real-world clinical practice scenario.

### Ultrasound assessment by specialists

Ultrasound images were independently evaluated by a radiologist experienced in thyroid pathology and an endocrinologist trained in cervical ultrasound. Each evaluator classified the nodules using the ACR TI-RADS (18) and Horvath TI-RADS systems (19) (the latter applied only by the expert radiologist), according to the original recommendations of each system. Evaluators were blinded to cytological results, to the recommendations issued by the artificial intelligence model, and to each other’s assessments.

### Artificial intelligence model

The convolutional neural network artificial intelligence model was previously trained at the University of Colorado, and it was designed for the analysis of thyroid ultrasound images (20). The model was applied without additional retraining, constituting an external validation in a real-world clinical environment. The training dataset included a heterogeneous spectrum of thyroid and non-thyroidal lesions, predominantly thyroid cancers with representation of different subtypes, but also lesions originating from non-thyroidal tissues, including 2 metastases from neuroendocrine tumors, 2 from chronic lymphocytic leukemia, 2 from breast carcinoma, 1 from colorectal carcinoma, as well as 1 parathyroid carcinoma and 1 thyroid lymphoma. For each nodule, researchers selected a representative transverse and longitudinal ultrasound image of the nodule, following predefined quality criteria (adequate focus, absence of artifacts, and complete visualization of the nodule), which was processed by the system to generate a recommendation regarding whether to perform FNA. Ultrasound image evaluators were blinded to cytological results and to each other’s classifications and recommendations.

### Reference standard

The reference standard was the cytological result obtained through fine-needle aspiration biopsy (FNA), classified according to the Bethesda System for Reporting Thyroid Cytopathology (21). For the analysis of clinical performance, nodules classified as Bethesda category III or higher were considered positive. This definition was used as an operational reference standard aligned with clinical decision-making for further diagnostic evaluation and management, rather than as a surrogate for definitive malignancy diagnosis. This approach reflects the study objective of evaluating FNA indication strategies in a real-world clinical context. Nodules classified as Bethesda I were excluded from the primary analysis due to their nondiagnostic nature.

### Statistical analysis

A descriptive analysis of clinical and ultrasound characteristics was performed. Sensitivity, specificity, positive and negative predictive values, and likelihood ratios were calculated for each biopsy indication method. These performance metrics were calculated based on the predefined operational reference standard (Bethesda ≥ III) and should be interpreted as measures of performance in identifying nodules requiring further diagnostic evaluation, rather than as indicators of diagnostic accuracy for malignancy. Agreement among the different methods (AI, radiologist, and endocrinologist) was assessed using Gwet’s AC1 coefficient. Analyses were performed using standard statistical software, with a significance level of p < 0.05. Given the retrospective nature of the study, no formal sample size calculation was performed.

## Results

### Study population

A total of 101 adult patients who underwent fine-needle aspiration biopsy (FNA) for thyroid nodules were included in the final analysis. The cohort consisted of 90 women (89.1%) and 11 men (10.9%), with a median age of 61 years (range: 18–88 years). The median nodule size was 2.3 cm (interquartile range [IQR]: 1.6–3.3 cm).

From an initial total of 122 biopsies, the cytological distribution according to the Bethesda System was as follows: 21 (17.2%) Bethesda I, 66 (54.1%) Bethesda II, 5 (4.1%) Bethesda III, 12 (9.8%) Bethesda IV, 2 (1.6%) Bethesda V, and 14 (11.5%) Bethesda VI. Bethesda I cases were excluded from the primary analysis. In the final cohort (n = 101), 33 nodules (32.7%) were classified as cytologically positive (Bethesda ≥ III), and 68 (67.3%) as cytologically negative (Bethesda II).

### Clinical performance of the evaluated strategies

The performance of the different FNA indication strategies, evaluated against the predefined cytological reference standard (Bethesda ≥ III) reflecting nodules requiring further diagnostic evaluation, is shown in [**Table 1**].

**Table 1:** Clinical performance metrics for FNA indication strategies (n = 101).

Diagnostic property	TI-RADS Horvath (expert radiologist)	ACR TI-RADS (expert radiologist)	ACR TI-RADS (expert endocrinologist)	AI
Sensitivity	0.82 (0.64–0.92)	0.85 (0.67–0.94)	0.82 (0.64–0.92)	0.88 (0.71–0.96)
Specificity	0.69 (0.57–0.79)	0.40 (0.28–0.52)	0.19 (0.11–0.31)	0.41 (0.30–0.54)
Positive Predictive Value (PPV)	0.56 (0.41–0.70)	0.41 (0.29–0.53)	0.33 (0.23–0.44)	0.42 (0.30–0.55)
Negative Predictive Value (NPV)	0.89 (0.76–0.95)	0.84 (0.66–0.94)	0.68 (0.43–0.86)	0.88 (0.70–0.96)
Positive Likelihood Ratio (LR+)	2.65 (1.79–3.91)	1.41 (1.11–1.79)	1.01 (0.83–1.23)	1.49 (1.18–1.89)
Negative Likelihood Ratio (LR−)	0.26 (0.13–0.55)	0.38 (0.16–0.90)	0.95 (0.40–2.28)	0.29 (0.11–0.77)

Cytological positivity was defined as Bethesda category III or higher (Bethesda ≥ III). Values are expressed as point estimates with 95% confidence intervals.

The Virtual Thyroid Biopsy (VTB) artificial intelligence model demonstrated a sensitivity of 0.88 (95% CI: 0.71–0.96) and a specificity of 0.41 (95% CI: 0.30–0.54). The Horvath TI-RADS system, evaluated by an expert radiologist, showed a sensitivity of 0.82 (95% CI: 0.64–0.92) and a specificity of 0.69 (95% CI: 0.57–0.79). The corresponding metrics for ACR TI-RADS, evaluated by expert radiologist and endocrinologist, as well as positive and negative predictive values and positive and negative likelihood ratios for each strategy, are detailed in **Table 1**.

### Distribution of recommendations according to cytological category

The distribution of FNA recommendations issued by the artificial intelligence model according to cytological category is described below. The model recommended FNA in all Bethesda V cases and in 13 of the 14 Bethesda VI cases. The model did not recommend FNA in one Bethesda VI case; however, biopsy was recommended for this case by both the Horvath TI-RADS and ACR TI-RADS systems.

The model recommended FNA for all 5 Bethesda III cases, of which 4 were confirmed as malignant in final pathology and 1 was lost to follow up. In the Bethesda IV category, the model recommended FNA in 9 of the 12 cases. Four of the 9 cases in which FNA was suggested underwent thyroidectomy and 3 were reported as malignant. Of the remaining 8 cases not referred for surgery, 7 underwent molecular testing, that reported low cancer risk and remained stable at ultrasound follow up, 1 patients was lost to follow up. Among Bethesda II nodules, the model recommended FNA in 39 of 66 cases, including the only patient with a confirmed cancer diagnosis in this category.

### Agreement between methods

Agreement between FNA recommendations issued by the artificial intelligence model and ultrasound classifications performed by human specialists is presented in **Table 2**. The percentage of agreement ranged between 60% and 63% across comparisons (p < 0.001). Gwet’s AC1 coefficient showed moderate agreement between the artificial intelligence model and ACR TI-RADS assessed by an expert endocrinologist (AC1 = 0.41; 95% CI: 0.22–0.61). Agreement between the model and ACR TI-RADS evaluated by an expert radiologist demonstrated an AC1 of 0.30 (95% CI: 0.09–0.51), while agreement with Horvath TI-RADS evaluated by an expert radiologist was 0.29 (95% CI: 0.09–0.48).

**Table 2:** Agreement between AI (VTB) and TI-RADS–based methods.

Method	Agreement (%)	95% CI	p-value	Gwet’s AC1	95% CI	p-value
ACR TI-RADS (Expert Endocrinologist) vs AI	0.63	0.54–0.73	<0.001	0.41	0.22–0.61	<0.001
ACR TI-RADS (Expert Radiologist) vs AI	0.60	0.50–0.70	<0.001	0.30	0.09–0.51	0.004
Horvath TI-RADS vs AI	0.63	0.54–0.73	<0.001	0.29	0.09–0.48	0.005

Agreement is expressed as proportion with 95% confidence interval. Concordance was evaluated using Gwet’s AC1 coefficient.

## Discussion

In this study, we evaluated the clinical performance of an artificial intelligence model to support the indication of fine‐needle aspiration biopsy (FNA) in thyroid nodules, comparing it with ultrasound classification systems used by human specialists in a real-world clinical setting. The results show that the AI model achieved performance metrics comparable to those of expert evaluators, particularly in terms of sensitivity, within a context characterized by operational heterogeneity and interobserver variability typical of public healthcare systems.

The widespread use of thyroid ultrasound in clinical practice, despite not being recommended as a systematic screening strategy, has contributed to a sustained increase in incidental detection of nodules and, consequently, in the indication of FNA, many of which correspond to benign lesions (3). In this context, variability in ultrasound interpretation represents a relevant challenge, even among trained specialists, which has led to the development of standardized systems such as TI-RADS (6, 15). However, previous studies have demonstrated that even with these systems, significant variability persists among evaluators, particularly in routine clinical scenarios (8).

The findings of this study suggest that the incorporation of AI-based tools may contribute to standardizing ultrasound interpretation and achieving greater homogeneity in FNA indication. Unlike previous studies that evaluated AI models in controlled settings or with prospectively selected images (11, 14) the present study examined the model’s performance under real-world clinical conditions, without additional retraining, and using images obtained during routine practice, thereby reinforcing its external validity (12, 13). An important consideration when interpreting these results is the trade-off between sensitivity and specificity observed in the AI model. While the model demonstrated high sensitivity, its lower specificity may lead to an increased number of biopsy recommendations. However, within the context of this study design, all included nodules had already undergone FNA. Retrospective application of the AI model would have resulted in 69 biopsy recommendations, potentially avoiding 32 procedures. This finding suggests that, when used as a triage support tool rather than in isolation, the model may still contribute to reducing unnecessary biopsies. These results highlight the importance of integrating AI outputs with clinical and radiological assessment to optimize decision-making and balance sensitivity with specificity in real-world practice.

When comparing the Bethesda category distribution observed in this study with international literature, some discrepancies were identified (19, 20). The proportion of non-diagnostic samples (17.2%) is higher than the ≤10% rate typically reported in highly specialized centers. However, this finding falls within the variability described in real-world clinical practice, where non-diagnostic rates may range between approximately 6% and 36% (22). This variability reflects known limitations of fine-needle aspiration, as sample adequacy may be influenced by multiple factors, including nodule characteristics (e.g., cystic or heterogeneous composition), variability in operator experience and cytopathological interpretation, and inherent constraints of cytological evaluation. Although both clot specimens and conventional cytology slides are routinely processed in our institution—an approach intended to improve sample adequacy—the observed rate likely reflects the multifactorial nature of cytological yield in routine practice.

Similarly, the lower proportion of benign results (54.1%) may be related to a selection bias toward higher-risk nodules, which may also explain the relatively high frequency of Bethesda VI (11.5%), higher than typically reported. In contrast, the overall proportion of indeterminate categories (13.9%) remained within the expected range (10–20% according to literature), suggesting adequate interpretive performance by the cytopathology laboratory despite variability in sampling conditions.

From a methodological perspective, the use of the Bethesda classification system as the reference standard represents an important limitation that should be carefully considered when interpreting the results. Bethesda cytology is useful for guiding clinical decision-making but does not constitute a definitive diagnosis of malignancy,particularly in indeterminate categories, as Bethesda III and IV. These categories are widely recognized as a major diagnostic challenge (23) due to their variable malignancy risk and limited discriminatory capacity. Bethesda ≥ III was defined as a positive result considering it an operational endpoint aligned with clinical decision-making for further diagnostic evaluation, rather than as a surrogate for confirmed malignancy. Accordingly, the performance metrics reported (including sensitivity, specificity, and predictive values) should be interpreted as measures of the ability of each method to identify nodules requiring additional work-up, rather than as estimates of true diagnostic accuracy for thyroid cancer. Importantly, not all nodules in our cohort underwent surgical resection, reflecting real-world clinical practice in which surgery is selectively indicated. As a result, histopathological confirmation was not available for all cases, limiting the ability to determine the true malignancy status across the entire cohort. Therefore, the reported accuracy metrics should be interpreted within the context of a cytology-based reference standard and a clinical decision-making framework. In this context, the exclusion of Bethesda I nodules was intended to avoid incorporating nondiagnostic results that do not meaningfully inform clinical decision-making or performance estimates.Although Bethesda I has traditionally been associated with a low malignancy risk, its nondiagnostic nature and variable risk estimates (0-20%) may introduce uncertainty and noise in performance analyses (21, 24). Cytology represents an appropriate reference standard when the clinical objective is to evaluate biopsy indication, it is important to acknowledge that cytology is not equivalent to definitive histopathology. In particular, indeterminate categories (Bethesda III and IV) are known to show significant discordance with final surgical outcomes, which may directly impact specificity, positive predictive value, and likelihood ratios of ultrasound-based risk stratification systems and artificial intelligence models as previously described in the literature (25).

**Figure 2:**
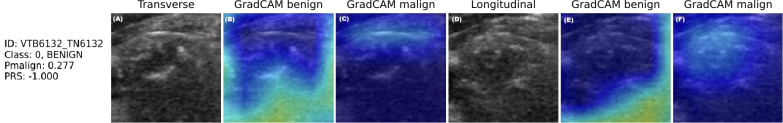
Representative ultrasound images and corresponding Grad-CAM saliency maps generated by the virtual thyroid biopsy (VTB) model. **(A)** transverse ultrasound image of the thyroid nodule. **(B)** Grad-CAM saliency map highlighting image regions contributing to the benign prediction in the transverse plane. **(C)** Grad-CAM saliency map highlighting image regions contributing to the malignant prediction in the transverse plane. **(D)** longitudinal ultrasound image of the same nodule. **(E)** Grad-CAM saliency map highlighting regions contributing to the benign prediction in the longitudinal plane. **(F)** Grad-CAM saliency map highlighting regions contributing to the malignant prediction in the longitudinal plane. The Grad-CAM maps illustrate the areas of the ultrasound images that most strongly influenced the model’s decision-making process. Warmer colors indicate image regions with higher contribution to the model’s prediction. In this example, the model classified the nodule as benign, with a malignancy probability of 0.277.

A single carcinoma case with Bethesda VI cytology (**Figure 2**) was observed in which the AI did not recommend biopsy, representing a clinically relevant but low-frequency false negative. In this case, FNA was recommended by ACR TI-RADS evaluated by both the radiologist and endocrinologist, as well as by Horvath TI-RADS. This case highlights that uncommon or atypical malignant phenotypes may not strongly activate AI decision thresholds. The nodule studied was a small oval, predominantly isoechoic nodule, containing subtle, ill-defined hypoechoic areas, and punctate echogenic foci suggestive of microcalcifications. The AI program has limitations in analyzing nodules smaller than 1 cm and the ill-defined margins represent a challenging feature for the program to recognize.

**Figure 3:**
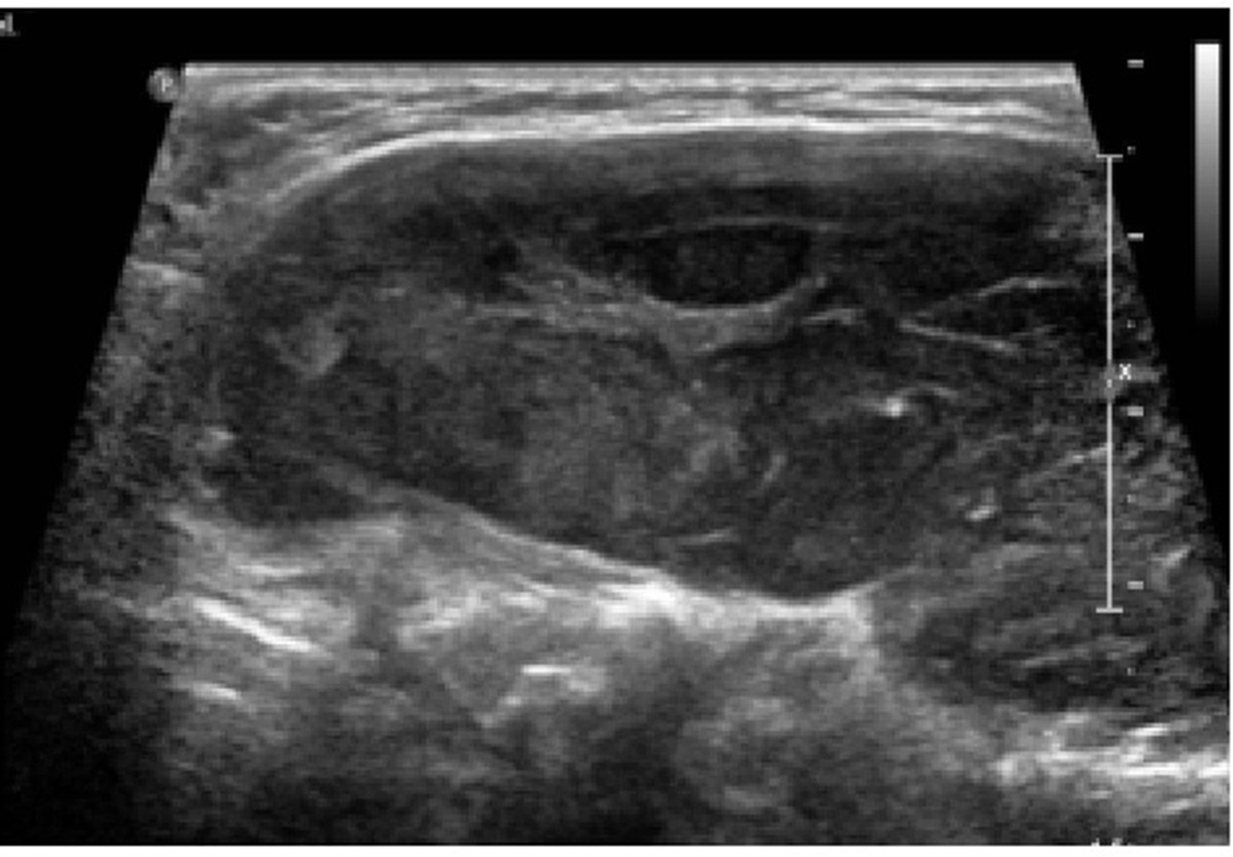
Thyroid ultrasound image of a nodule subsequently diagnosed as lymphoma. Image shows diffuse enlargement of the thyroid gland, characterized by a markedly heterogeneous and hypoechoic echotexture with smooth margins. No discrete thyroid nodule can be clearly identified.

Notably, the model correctly identified a case that was subsequently diagnosed as lymphoma (**Figure 3**). The initial FNA cytology was reported as Bethesda II. In this scenario, both the AI model and the different methods applied by human specialists recommended performing FNA, which prompted progression to a core biopsy and allowed confirmation of the final diagnosis. This case illustrates the inherent limitations of any rigid operational definition and highlights the irreplaceable role of clinical judgment in interpreting cytological results and guiding subsequent management decisions. Importantly, the AI model was able to successfully overcome this challenge.

In summary, the analysis of discordant cases highlights inherent limitations in both AI models and traditional ultrasound classification systems. The case in which the model did not recommend FNA for a nodule with Bethesda VI cytology, as well as the thyroid lymphoma case, reflect complex clinical scenarios in which ultrasound characteristics may not conform to typical patterns. These findings reinforce the need to interpret recommendations generated by automated tools as support for clinical decision-making rather than as substitutes for comprehensive clinical judgment.

Importantly, the present study was designed to evaluate performance in the clinical decision-making process for fine-needle aspiration recommendation, rather than to assess definitive diagnostic accuracy for thyroid malignancy. Therefore, direct comparison with studies using surgical histopathology as the reference standard should be interpreted with caution, as these approaches address different clinical questions.

This study has several relevant strengths, including the evaluation of an AI model in a real-world clinical practice in a resource limited setting, direct comparison with human specialists, and the use of robust agreement metrics such as Gwet’s AC1 coefficient. However, certain limitations must also be acknowledged. Its retrospective design and convenience sampling may introduce bias, and the relatively small sample size restricts the generalizability of the findings.

From a methodological perspective, the interpretation of sample size in diagnostic accuracy studies should not rely solely on the total number of participants, but also on the distribution of cases and controls used to estimate sensitivity and specificity. As highlighted by Flahault et al. (26), study design and interpretation should be guided by the expected precision of these metrics rather than by sample size alone.

In the present cohort, the inclusion of 33 cytologically positive nodules and 68 negative cases enabled estimation of key performance metrics in a real-world clinical setting. The width of the confidence intervals, while reflecting some degree of uncertainty, remained sufficiently narrow to allow clinically meaningful interpretation of the results, particularly for sensitivity estimates, given the number of positive cases available.

Given the retrospective nature of the study, no formal sample size calculation was performed. Nevertheless, the relatively limited sample size may still affect the precision of the estimates and the stability of certain metrics, particularly specificity, and therefore the findings should be interpreted as exploratory and hypothesis-generating. Further prospective, multicenter studies with larger sample sizes are required to confirm these results.

Overall, these results suggest that artificial intelligence models may play a complementary role in the ultrasound evaluation of thyroid nodules, particularly as decision-support tools for FNA indication in high-demand healthcare settings with limited specialist availability. Prospective multicenter studies with larger sample sizes are necessary to further assess their clinical impact and safe integration into routine clinical workflows.

## Conclusion

In a real-world clinical practice setting, the evaluated artificial intelligence model demonstrated clinical performance comparable to that of human specialists for indicating fine-needle aspiration biopsy (FNA) in thyroid nodules. Its sensitivity metrics and agreement with commonly used ultrasound classification systems suggest that such tools may contribute to clinical decision-making and help reduce interobserver variability. However, their use should be understood as complementary to clinical judgment, and future prospective studies will be necessary to determine their impact on clinical practice.

## Data Availability

The data analyzed in this study is subject to the following licenses/restrictions: Information contained in the dataset are not publicly available due to ethical and legal restrictions related to patient confidentiality. All data were anonymized and coded prior to analysis, and no identifiable patient information was shared. Access to the data is restricted in accordance with institutional and ethical approval requirements. Requests to access these datasets should be directed to Corresponding author, Dr. Cristina Goens (mcristina.goens@hospitallaflorida.cl). Access may be granted upon reasonable request and subject to institutional ethical approval and data protection regulations.
